# High resolution biologging of breaching by the world’s second largest shark species

**DOI:** 10.1038/s41598-021-84670-3

**Published:** 2021-03-04

**Authors:** Jessica L. Rudd, Owen M. Exeter, Jackie Hall, Graham Hall, Suzanne M. Henderson, Christopher Kerry, Matthew J. Witt, Lucy A. Hawkes

**Affiliations:** 1 Hatherly Laboratories, College of Life and Environmental Sciences, University of Exeter, Prince of Wales Road, Exeter, EX4 4PS UK; 2 Environment and Sustainability Institute, University of Exeter, Penryn Campus, Cornwall, TR10 9FE UK; 3 Centre for Ecology and Conservation, University of Exeter, Penryn Campus, Cornwall, TR10 9FE UK; 4 Manx Basking Shark Watch, Glen Chass Farmhouse, Glen Chass, Port St Mary, Isle of Man IM9 5PJ UK; 5 NatureScot, Great Glen House, Inverness, Scotland IV3 8NW UK

**Keywords:** Biomechanics, Behavioural ecology, Biooceanography, Conservation biology

## Abstract

Basking sharks, the world’s second largest fish, are endangered globally following two centuries of large-scale exploitation for their oily livers. In the northeast Atlantic, they seasonally gather in key sites, including the western Scottish Isles, where they feed on plankton, but their breeding grounds are currently completely unknown. Using high-resolution three-axis accelerometry and depth logging, we present the first direct records of breaching by basking sharks over 41 days. We show that basking sharks breach both during the night and day, starting at approximately 20 m depth and can breach multiple times in short succession. We also present early evidence of potential lateralisation in basking sharks. Given the energetic nature of breaching, it should have an important biological function, but this remains unclear.

## Introduction

The emergence of animal tracking technologies (‘biologging’) over the past 50 years has provided invaluable insight into movements, behaviour, and energetics of a wide variety of species^1,2^. Basking sharks (*Cetorhinus maximus*) are the world’s second largest fish, reaching up to 12 m in length and four tonnes in weight. Following two centuries of large-scale exploitation for their oily livers^3^, they are considered endangered globally. Their long-distance and seasonal movements in the northeast Atlantic have been described^4–6^, as well as their habitat preferences^7^ and diving patterns^8^, but many aspects of their reproductive behaviour remain enigmatic, and their breeding grounds are still completely unknown^3^. One of the world’s largest conspicuous seasonal foraging aggregations of basking sharks forms in the summer off the west coast of Scotland, in the Sea of Hebrides^4,7–9^. While sharks likely visit there to feed, these aggregations may also provide opportunity for social interactions and courtship. Breaching (leaping entirely, or almost entirely out of the water) has been noted in basking sharks in the west coast of Scotland^9^ along with courtship-like behaviours such as close following, parallel and echelon swimming^9,10^. Johnston et al.^10^ estimated that basking sharks can breach at greater vertical speeds than predatory ambushes performed by great white sharks, requiring as much as 5 to 6% of their daily standard metabolic rate, estimated from a single recorded breaching event. Given the energetic investment required to breach it seems plausible that this behaviour has a fitness benefit, such as securing a mate.

## Results & discussion

**Figure 1:**
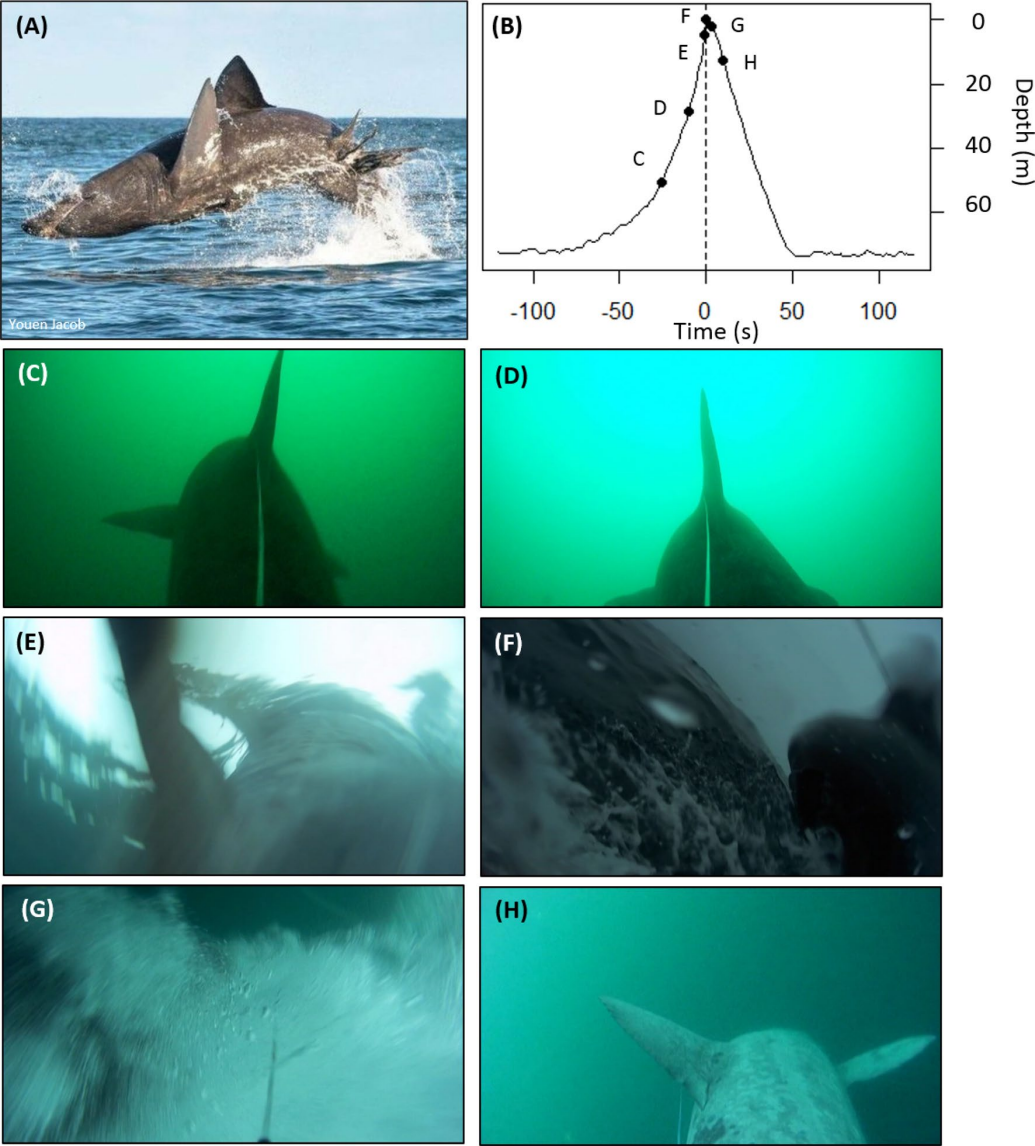
Basking shark breaching. Breaching recorded by a towed camera tag deployed in 2018. These data are from a shark that was not instrumented with an accelerometer, they are included to aid visualisation of the breaching process from a point-of-view perspective. For sharks instrumented with accelerometers in 2017, tags where attached flush to the surface of the animal at the base of the dorsal fin. (**A**) Basking shark breaching (photo: Youen Jacob). The timing and depth associated with each image (**C**–**H**) are identified on the breaching depth profile (**B**). (**C**,**D**) the shark starts to ascend from 72 m depth at 0.94 m of vertical gain per second, reaching the surface (in view, **E**) in 77 s. The shark can be seen completely out of the water (**F**), before descending (**G**,**H**) to depth again.

**Figure 2:**
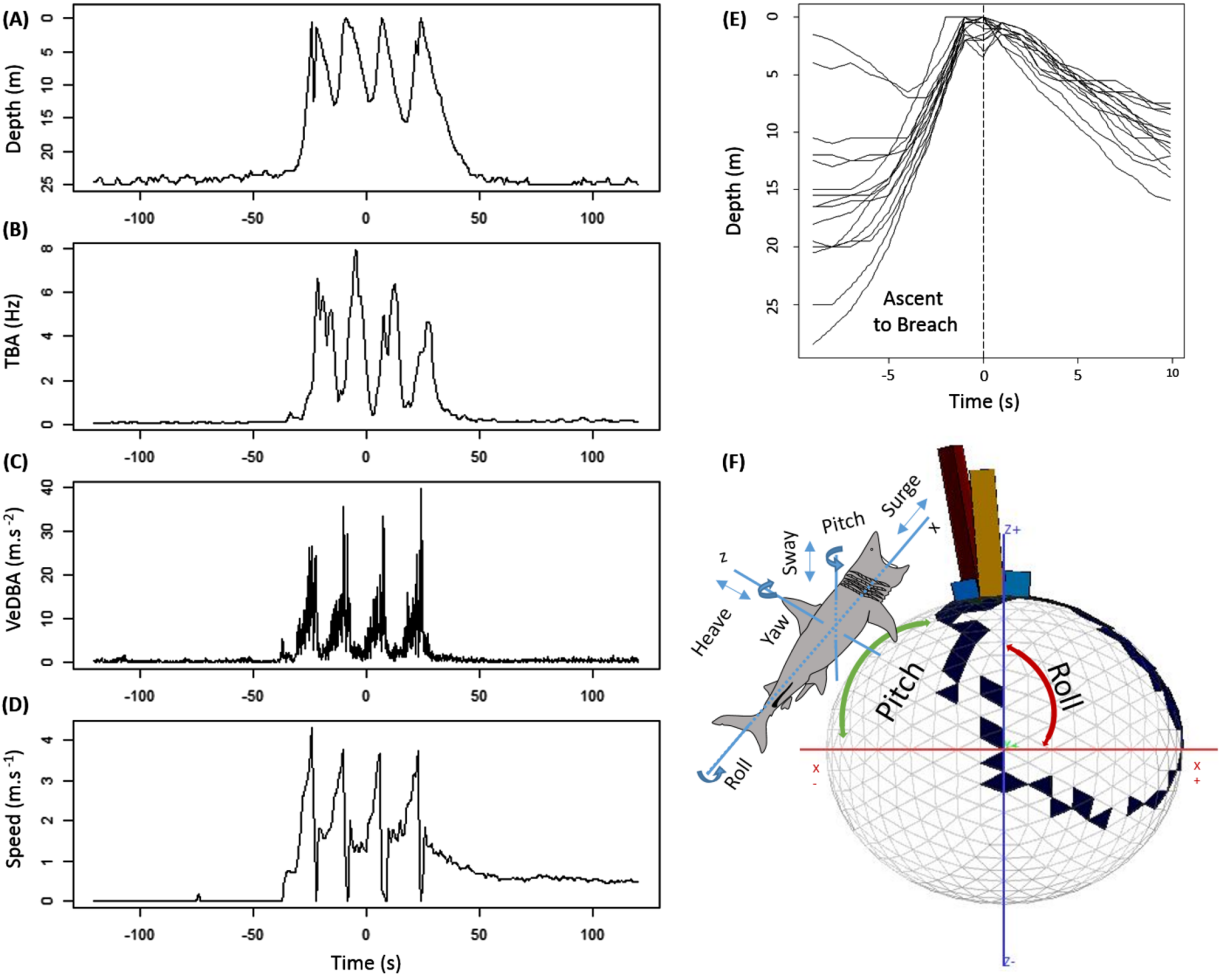
Characteristics of breaching. (**A**–**D**) A quadruple breach by a six-metre basking shark over 47 s showing changes in depth (**A**), tail beat amplitude (**B**), VeDBA (**C**) and speed (**D**) over the series of breaches. (**E**) Depth profiles of 16 single breaching events performed by a single shark, with time (in seconds) centred on the breach, overlaid on a common timescale showing repeatability of ascent angle and subsequent descent after breaching. (**F**) Dubai plot showing tri-axial acceleration data as a 3-dimensional histogram, with time spent by sharks in a particular posture on each facet of the sphere extruded as triangular bars, and colour scaling with the cumulative time in a given facet. Data show a right-handed breach of a single shark, where rapid rolling is indicated by short dark blue bars on the right face of the sphere.

In the present study, we used accelerometer enabled animal-borne biologging tags (recording temperature, pressure and three-axis accelerometry) to describe in high temporal resolution the variability and repeatability of 67 breaches made by three sharks over 41 cumulative days (Fig. 1; shark 5 m length (n = 1) 678 kg estimated mass; and sharks 6 m length (n = 2) 160 kg estimated mass). Approximately half (n = 28) of all breaches were single breaches, but we also recorded 13 double breaches, three triple breaches and one shark that breached four times in 47 s (Fig. 2A-D). Consecutive breaches were 18 s apart (mean value ± 6 s.d, range 12–47 s); i.e. sharks ascend from depth to the surface, propel themselves out of the water and swim to depth before commencing the subsequent ascent. Breaching frequency varied among individuals. Shark 1 breached 0.4 ± 0.9 times per day (mean ± 1 s.d.; range 0–2, n = 2 breaches), shark 3 breached 0.9 per day (± 0.9 s.d, range 0–2, n = 5) and shark 2 breached 1.9 times per day (± 1.8 s.d, range 0–6, N = 60) over 4.8, 4.9 and 31.8 tracking days respectively), during both day and night (peak hour of breaching 04:00 am). Multiple breaches by the same sharks have never been empirically demonstrated before, and breaching has not been described to take place at night.

At the onset of a breach, sharks switched from slow swimming at 0.3 m.s^-1^ (mean value ± 0.16 s.d., range 0.17–0.4 m.s^-1^) at 14.8 m depth (± 5, range 4.6–28 m), to swimming towards the surface (Fig. 1C-E) at an angle of 38.9° (± 13.2, 23.08 to 81.6°), and an average (mean) ascent speed of 2.7 m.s^-1^ ± 0.5 (1.2–3.8 m.s^-1^). The peak ascent phase of a breach was observed when the rates of ascent and swimming speed rapidly increased. Breaching metrics were calculated separately for this peak ascent phase, where basking sharks reached the surface in 6 s (± 2.1, 2–17), before breaching near vertically at 76° (± 9, 43.3–87.9°), leaving the water at a mean exit speed of 3.9 m.s^-1^ ± 0.6. range: 2.2–5.6 m.s^-1^) (Fig. 1F). To contextualise our observations, an 8 m basking shark breached at 5.1 m.s^-1^ from 28 m depth^10^ and oceanic whitetip sharks (*Carcharhinus longimanus*)^11^ and great white sharks (*Carcharodon carcharias*)^11,12^ ambush-breach their prey at 4 and up to 6.5 m s^−1^ respectively, but from considerably deeper (> 100 m^11^) and are smaller sharks. The peak forces generated by the three tagged basking sharks (which were estimated to weigh up to 1160 kg) were 20 G at the peak of breaching. Breaches could be further characterised by whether sharks exited the water on a particular side of their body. Sharks rolled to their right side in 45 of the 67 breaches (representing 67% of breaches), which may be suggestive of lateralisation (Fig. 2F), the preference for breaching on one side consistently across events^13,14^. Dynamic body acceleration (VeDBA) (linear mixed effects model; χ^2^ 7.6, p = 0.006) along with tailbeat amplitude (linear mixed effects model; χ^2^ 5.54, *p* = 0.019) increased with the sharks’ ascent pitch towards the surface. Breaching events were highly repeatable, both among and between sharks, following a similar ascent rate, speed and angle, and from a similar starting depth (Fig. 2E). Breaching was more energetically demanding than routine swimming (breaching VeDBA 7.7 m s^−2^ ± 4.5, range 0.4–14.7 vs routine swimming VeDBA 0.24 m.s^-2^ ± 0.04, 0.2–0.27), requiring double the tail beat frequency (breaching 0.49 Hz ± 0.12 vs routine swimming 1.08 Hz ± 0.51) and 15 times the tail beat amplitude (breaching 1.5 ± 1.1 Hz vs. routine swimming 0.1 ± 0.05 Hz). During multiple breaching events, the ascent rate, swimming speed and acceleration were similar for every subsequent breach, although the ascent starting depth was often shallower than for the initial breach. The relatively low field metabolic rate that comes with being ectothermic makes energetically demanding behaviour relatively more expensive for sharks. Therefore, the costs of performing multiple breaches may accumulate more rapidly compared to endothermic whales, such as humpback whales (*Megaptera novaeangliae*), which have been recorded breaching 17 times in a 6.5 h deployment^15^. On average, sharks required an estimated 11.5 kJ (range 3–22 kJ) of mechanical energy (${E}_{m}$) to perform a breach, and expended the same ${E}_{m}$ for each breach, regardless of whether they breached once or several times (Wilcoxon rank sum test, W = 198.5, p = 0.87; ${E}_{m} single$ = 11.5 to 11.8 kJ, ${E}_{m} multi$ = 9.98 to 10.3 kJ). Comparatively, the energetic cost of breaching for an 8 m basking shark weighing 2700 kg was estimated six times greater (63 to 72 kJ^10^). These differences may be attributed to the sharks in the present study being smaller, with the cost of breaching found to increase with increasing body mass^15^. A breach likely constitutes approximately 0.05 to 0.09% of their daily metabolic cost, which ranged from 12.8 to 21.5 MJ per day^16^. For comparison, the relative cost of performing a single breach in a 7.8 m (7000 kg) humpback whale represents 0.08 to 0.5% of its daily field metabolic rate^15^.

The question remains what the function of breaching is for basking sharks. We are still far from certain what the function of breaching is for many aquatic species, but spinner dolphins, blacktip sharks and humpback whales are known to breach to dislodge epiparasites^17^. Gore et al.^9^ noted that epiparasitic lampreys were not dislodged from basking sharks following breaching, suggesting that it may not function for parasite removal, or may require several breaches to dislodge such parasites. Breaching may be used to visually signal between spinner dolphins, and between humpback whales^17^. Basking sharks breached during the night-time as well as the daytime, and have small eyes, suggesting that breaching is unlikely to be a visual signal. However, breaching may play a role in acoustic communication between distant groups of sharks. Basking sharks can apparently detect weak electric signals produced by zooplankton^18^, and some elasmobranchs use electro-sensory cues during courtship^19^, suggesting that breaching could convey readiness to mate. It thus seems possible that the acoustic signal of breaching could be detectable and useful to basking sharks. We have no information in the present study about the presence of other sharks during breaching, although future work using animal-borne acoustic proximity receivers on large numbers of sharks, or aerial drones, could provide insight into the social networks of basking sharks, and whether they breach in proximity to conspecifics. We propose that in the absence of a better explanation and given the predictable and persistent aggregations of basking sharks in Scottish waters, that breaching may be more likely to be related to intra-specific signalling, than anything else yet described.

We show using repeated direct measurements from three individuals, that the mechanical forces required for basking sharks to breach are considerable, but that basking sharks can breach repeatedly in quick succession. The role of breaching seems most likely to be related to intra-specific signalling and may add to a weight of evidence suggesting that Scottish waters may be an important site for breeding for the species.

## Methods

All work was carried out in accordance with the UK HM Government Home Office under the Animals (Scientific Procedures) Act 1986 (Licence P23C6EFD2) and under the Wildlife & Countryside Act 1981 (as amended) (Licence: 124,812), and were reviewed and approved by the University of Exeter’s Animal Welfare and Ethical Review Board (AWERB). Three basking sharks (two females, one unidentified sex determined by a sub-surface video camera system) were tagged from a Nelson 42 vessel using a tagging pole in the waters of Coll and Tiree, Inner Hebrides, Scotland (N 56° 33′, W 6° 41′) with Daily Diary tags (“DD tags”, TDR10-DD-278A, Wildlife Computers, WA, USA) placed at the base of the left-side of the primary dorsal fin, between 2nd August and 4th September 2017. Based on body length maturity estimates^3^, these two female sharks were juveniles. DD tags recorded accelerometry at 8 Hz on three orthogonal planes corresponding to the dorso-ventral, anterior–posterior and lateral axes of the animal. Accelerometry data are comprised of i) static (low-frequency) acceleration obtained by smoothing each of the acceleration measurements of acceleration in the X,Y and Z dimensions respectively with a running mean of three seconds [S1, S2], and ii) dynamic (high-frequency) acceleration, obtained by subtracting the static acceleration from the raw data for the corresponding X, Y and Z axes and used to calculate the Vectorial Body Dynamic Acceleration (VeDBA, see also Supplementary Materials). Breaching events were identified by a wet/dry sensor (recording at 4 Hz) as events where the tag broke the surface of the water. Data were individually inspected, looking for rapid depth changes coinciding with peaks in dynamic acceleration to identify breaching events. For each breach, the ascent and descent phases of breaching events were described by changes in depth, VeDBA, speed and tail beat amplitude, including the maximum and absolute mean pitch and roll angles, the ascent and descent depths and duration. The ascent phase began when the sharks made a directed, sustained swim to the surface and usually terminated in a shorter, peak ascent phase, where the period of burst speed ≥ 1.5 m.s^-1^ before breaching. The descent phase was the period between the end of the breach until the sharks’ depth levelled. See also Supplementary Materials.

## Supplementary Information

Supplementary Information 1.

Supplementary Video 1.

## Data Availability

The datasets generated during and/or analysed during the current study are available from the corresponding author on reasonable request.
