# Supplementary material for: High resolution biologging of breaching by the world’s second largest shark species
Source: Sci Rep. 2021 Mar 4;11:5236. doi: 10.1038/s41598-021-84670-3 (PMC7933335; doi:10.1038/s41598-021-84670-3)
Supplement: Supplementary file 1 — Supplementary Information 1. [file 41598_2021_84670_MOESM1_ESM.docx]

**Supplementary experimental procedures**

**High resolution biologging of breaching by the world’s second largest shark species**

Jessica L. Rudd, Owen M. Exeter, Jackie Hall, Graham Hall, Suzanne M. Henderson, Christopher Kerry, Matthew J. Witt, Lucy A. Hawkes

**Study area and tagging**

Three basking sharks measuring 5 and 6 m in length (two females, one unidentified sex, determined by a sub-surface video camera system) were tagged in the waters of Coll and Tiree, Inner Hebrides, Scotland (N 56˚33’, W 6˚41’) with Daily Diary tags (“DD tags”, TDR10-DD-278A, Wildlife Computers, WA, USA) between 2^nd^ August and 4^th^ September 2017. DD tags recorded accelerometry and magnetometry at 8 Hz on three orthogonal planes corresponding to the dorso-ventral, anterior-posterior and lateral axes of the animal. DD tags also recorded swim speed (m.s^-1^), depth (m) and temperature (C˚) at 1 Hz and whether the tag was wet or dry using a salt water switch sensor recording at 4 Hz. Tags were attached using a custom-made darting system to the body at the base of the dorsal fin that held the tags flush to the body (Fig. S1). A programmable timed-release mechanism (Wildlife Computers, WA, USA) released the tag from the shark 30 days following attachment. Each tag was deployed with a hydrodynamic syntactic bead floatation system (AZ-FLOAT-006, Wildlife Computers, 16.9 x 4.1 x 10.5 cm, 80 g in air) and a SPOT6 satellite tracking tag (Wildlife Computers) to aid relocation of DDs once tags had detached from the study animals. Each tag system also comprised of a second satellite tag, a SPOT5 towed tag, which tracked the sharks’ movements during the DD-tag deployment, and remained on the sharks after the DD tag detached. Following detachment, DD tags were retrieved and data downloaded.

**Accelerometry and behavioural analysis**

There were small differences in the angles at which tags were deployed on sharks, so to correct the orientation of the tags to the sharks’ body axes, accelerometry data were calibrated (following rotation of known angles) using the ‘tagtools’ package (<https://github.com/stacyderuiter/TagTools>). Accelerometry data were calibrated to one unit of gravity (9.8 m.s^-1^) by rotating the DD through known angles in all three spatial planes. The sharks’ body pitch and roll were extracted from the raw accelerometry data using additional ‘tagtools’ functions, with positive and negative angles indicative of an upward and downward pointing direction, respectively. Temperature and depth data were linearly interpolated to match the accelerometry and magnetometry 8 Hz sampling frequency. Swimming speeds for shark 3 were omitted due to likely entrapment of material preventing rotation of the speed wheel, making the data quality poor. Magnetometry data were omitted due to the metal base-plate on which the DD-tags were attached that caused false readings by the magnetometer.

Accelerometry data comprises two components, (i) low-frequency static acceleration and (ii) high-frequency dynamic acceleration. The static component relates to the inclination of the tag with respect to the earth’s gravitational field (which is analogous to the shark’s body posture) and was obtained by individually smoothing each of the three acceleration channels with a running mean of three seconds [S1, S2]. These smoothed values were then subtracted from the raw data for the corresponding axis, leaving three-dimensional dynamic acceleration, relating to the changes in velocity owing to the patterns of the animal’s movement [S3]. The three-dimensional dynamic acceleration was then used to make a summary metric describing effort, VeDBA (Vectorial Body Dynamic Acceleration) calculated as follows:

$VeDBA=\sqrt{(A_{x}^{2}+A_{y}^{2}+A_{z}^{2})}$ (1)

VeDBA is considered a proxy for the rate of energy expenditure, when tag orientation varies over time [S4, S5].

Mean swimming speed, VeDBA, depth and tail beat amplitude (TBA – see below, hereafter referred to as ’swimming metrics’) were compared for diel differences between sunrise and sunset times for the study site (<https://www.tidetimes.org.uk/gott-bay-tide-times>).

A spectrogram of the dorso-ventral acceleration (z-axis) was generated in Ethographer ver. 2.04 [S6] in Igor Pro (Igor Pro 8, WaveMetrics Inc., Lake Oswego, USA), calculated by continuous wavelet transformation using the Marlet wavelet function with a minimum cycle of 0.125 seconds and maximum cycle of 1 second [S6]. TBA was calculated for each 1 second interval using the Peak Tracer function. The resulting values were linearly interpolated to match the 8 Hz sampling frequency of the Daily Diary, as above.

**Energetics**

The morphometric measurements of shark appendages, fork length and mass were calculated as described in [S7]. Shark 1 was estimated to weigh 678 kg and measure 5 m, while sharks 2 and 3 were both estimated to weigh 1160 kg and measure 6 m. To derive the mechanical power of each breach (E_m_), expressed in kJ described in [S7], as:

$E_{m}=\frac{k_{E}}{ƞ_{k}}.\frac{{mv}^{2}}{2}$ (2)

where $k_{E}$ is a parameter likely ranging between 1.3 and 1.5 reflecting the acceleration profile of the shark during a breach [S7] , the hydrodynamic propulsion efficiency $ƞ_{k}$ is estimated at 0.7, the mass of the shark *m* in kg, and the speed of shark *v* in m.s^-1^ (see S7 for calculations). Since the speed *v* was derived from the speed wheel, the mechanical power of breaches could only be calculated for sharks 1 and 2. E_m_ was estimated for both the 1.3 and 1.5 values of $k_{E}$ to derive the range of mechanical power of each breach.

Sharks’ daily energy expenditure was estimated using mean daily summed VeDBA, and compared to an estimate of daily routine metabolism (in kJ.hr^-1^) using a generalised relationship between mass and routine metabolism for 17 species of fish, including five shark species, given in [S8]. The wet mass of copepods required to meet the sharks daily energetic expenditure was calculated from the calorific value of copepods of 5.04 kJ.g^-1^ [S9]. Since zooplankton densities were not measured *in situ* for the present study, a prey concentration range was used to estimate the amount of time necessary for the sharks to filter-feed the amount of prey needed to meet energetic demand, where the minimum density was the average theoretical threshold (0.62 g.m^-3^) for prey concentration estimated in [S10], while the maximum density was 2.41 g.m^3^ as reported in the same paper.

In the present study, as foraging events could not be identified from accelerometry data, the filtration rates for both the 5 m and 6 m sharks were derived from [S10] estimates and adjusted to the average swimming speed recorded by the DD tags (0.4 m.s^-1^ and 0.2 m.s^-1^ respectively). While the gape area of a 5 m shark was reported at 0.2 m^2^ in [S10], it was estimated at 0.3 m^2^ for a 6 m shark based on the 1:10 gape length – total body length ratio [S10], with resulting filtration rates of 207 m^3^.h^-1^ and 123 m^3^.h^-1^ respectively (Fig. S3).

**Postural data analysis**

To describe changes in the sharks posture during particular behaviours, the static acceleration of all three acceleration axes were plotted in a three dimensional scatter plot (hereafter termed a ‘g-sphere’) [S11]. The shark’s body orientation is represented by the position on the g-sphere, with the “north pole” (top of the sphere) denoting a horizontal swimming posture. All deviations from the north pole represent different orientations of the shark relative to its horizontal position. The ‘Dubai plot’ function generates a three-dimensional histogram of the frequency of postural data in each facet of the sphere, with the height of each bar equivalent to the total time spent by the shark in the corresponding posture. The posture of the shark during breaching was then quantified visually as breaching forward, backwards, to the left or right side. The last three breaches made by shark 2 (5%) were disregarded in the analysis due to potential shifts in the position of the DD tag and providing likely misleading postural data.

**Statistical analysis**

To identify whether breaching occurred at particular times of the day, the frequency distribution of breaches throughout the 24-hour cycle was tested using Rayleigh test of uniformity with the R package ‘CircStats’ (<https://cran.r-project.org/web/packages/CircStats/CircStats.pdf>). Relationships between VeDBA and both ascent pitch and tail beat frequency were examined using linear mixed effect models in R (package: lme4) with a random effect of individual to account for repeat observations from multiple individuals. Statistical differences between variables describing the ascent and descent phases of breaching events (depth, VeDBA, TBA, speed, pitch and roll angles) were tested with paired t-tests or Wilcoxon signed-rank test depending on normality. Breaching may be expected to be energetically expensive, and multiple breaches to be progressively more demanding. Thus, to test whether breaching multiple times was different to breaching just once, swimming metrics (changes in depth, VeDBA, Speed and TBA, maximum and absolute mean pitch and roll angles, the starting ascent and finishing descent depths, and dive duration) were compared using paired t-tests or Wilcoxon signed-rank test depending on whether they met assumptions of normality. Lateralisation, the preference for one side (left or right) that is consistent across events [S12], was investigated by comparing the roll and pitch direction during breaching. Due to limited sample size of breaching events for sharks 1 and 3 (n=2 and n=5 respectively) only sideways breaches made by shark 2 were included in the lateralisation analysis. Chi-squared test with Yates continuity correction were used to test for the association between direction of the roll and diel period.

**Supplementary materials for this manuscript include the following:**

**Video S1**: Video of basking shark breaching gathered from a towed camera system deployed in 2018. These tags require the use of tethers that were not required for accelerometers deployed in 2017, which were attached flush to the body of sharks. Perceived rotation observed of the shark is an artefact of a swivel used in the flexible tether system, the sharks themselves do not rotate during ascent.

**Supplemental references**

S1. Wilson, R. P. *et al.* Moving towards acceleration for estimates of activity‐specific metabolic rate in free‐living animals: the case of the cormorant. *J. Anim. Ecol*. **75(5),** 1081-1090 (2006).

S2. Shepard, E. L. *et al.* Identification of animal movement patterns using tri-axial accelerometry. *Endanger. Species Res*. **10**, 47-60 (2008).

S3. Gleiss, A. C., Wilson, R. P., & Shepard, E. L. Making overall dynamic body acceleration work: on the theory of acceleration as a proxy for energy expenditure. *Methods Ecol. Evol*. **2(1)**, 23-33 (2011).

S4. Qasem, L. *et al.* Tri-axial dynamic acceleration as a proxy for animal energy expenditure; should we be summing values or calculating the vector?. *PloS one*. **7(2),** e31187; 10.1371/journal.pone.0031187 (2012).

S5. Wright, S., Metcalfe, J. D., Hetherington, S., & Wilson, R. Estimating activity-specific energy expenditure in a teleost fish, using accelerometer loggers. *Mar. Ecol. Prog. Ser.* **496**, 19-32 (2014).

S6. Sakamoto, K. Q. *et al.* Can ethograms be automatically generated using body acceleration data from free-ranging birds?. *PLoS one*. **4(4)**, e5379; 10.1371/journal.pone.0005379 (2009).

S7. Johnston, E. M. *et al.* Latent power of basking sharks revealed by exceptional breaching events. *Biol. Lett.* **14(9),** 20180537; 10.1098/rsbl.2018.0537 (2018).

S8. Parsons, G. R. Metabolism and swimming efficiency of the bonnethead shark *Sphyrna tiburo*. *Mar. Biol.* **104(3),** 363-367 (1990).

S9. Båmstedt, U. Chemical composition and energy content in *The biological chemistry of marine copepods* (eds. Corner, E. D. S., & O'Hara, S. C. M.), (Oxford University Press, USA, 1986)

S10. Sims, D. W. Threshold foraging behaviour of basking sharks on zooplankton: life on an energetic knife-edge?. *Proc. R. Soc. Lon. Series B*. **266(1427),** 1437-1443 (1999).

S11. Walker, J. S. *et al.* Prying into the intimate secrets of animal lives; software beyond hardware for comprehensive annotation in ‘Daily Diary’ tags. *Mov. Ecol.* **3(1),** 1-16 (2015).

S12. Canning, C. *et al.* Population-level lateralized feeding behaviour in North Atlantic humpback whales, *Megaptera novaeangliae*. *Anim. Behav*. **82(4),** 901-909 (2011).
